# Evolutionary insights into Interleukin-12 family targets across 405 species

**DOI:** 10.3389/fimmu.2025.1584460

**Published:** 2025-05-30

**Authors:** Weibin Wang, Dawei Li, Kaiyong Luo, Baozheng Chen, Xuzhen Li, Tingting Hao, Dazhong Guo, Yang Dong, Ya Ning

**Affiliations:** ^1^ College of Science, Yunnan Agricultural University, Kunming, Yunnan, China; ^2^ Yunnan Provincial Key Laboratory of Biological Big Data, Yunnan Agricultural University, Kunming, Yunnan, China; ^3^ Pain Management, The Second Affiliated Hospital of Kunming Medical University, Kunming, Yunnan, China

**Keywords:** interleukin-12, cytokine evolution, targeted therapy, cancer, Metazoa

## Abstract

The Interleukin-12 (IL-12) family ligand subunits (IL-12s) and receptor subunits (IL-12Rs) constitute pivotal regulators of immune homeostasis, with direct implications in autoimmune pathologies and oncogenesis. Through phylogenetic reconstruction, synteny analysis, and sequence alignment across 400+ animal species, we delineated the evolutionary trajectories and functional diversification of these immune mediators. Phylogenetic analysis revealed IL-12Rs originated prior to the mollusk era (514-686.2 million years ago, Mya), while ligand subunits p19/p28 emerged during the mammalian and avian epoch (180-225 Mya). Structural characterization identified three evolutionarily invariant signature motifs within the fibronectin type III (fn3) domain essential for receptor-ligand interface stability. Furthermore, phylogenetically ultra-conserved residue and motif configurations were mapped, revealing candidate therapeutic epitopes. These findings establish an evolutionary framework explaining functional conservation/divergence in IL-12 signaling components. The identified ancient receptor architectures coupled with derived ligand innovations provide a blueprint for cross-species immunotherapy design targeting conserved interaction interfaces. The conserved molecular signatures offer dual utility in developing precision therapies and interspecies disease management strategies, particularly for translational applications across human medicine, agriculture, and aquaculture.

## 1 Introduction

**Figure 1:**
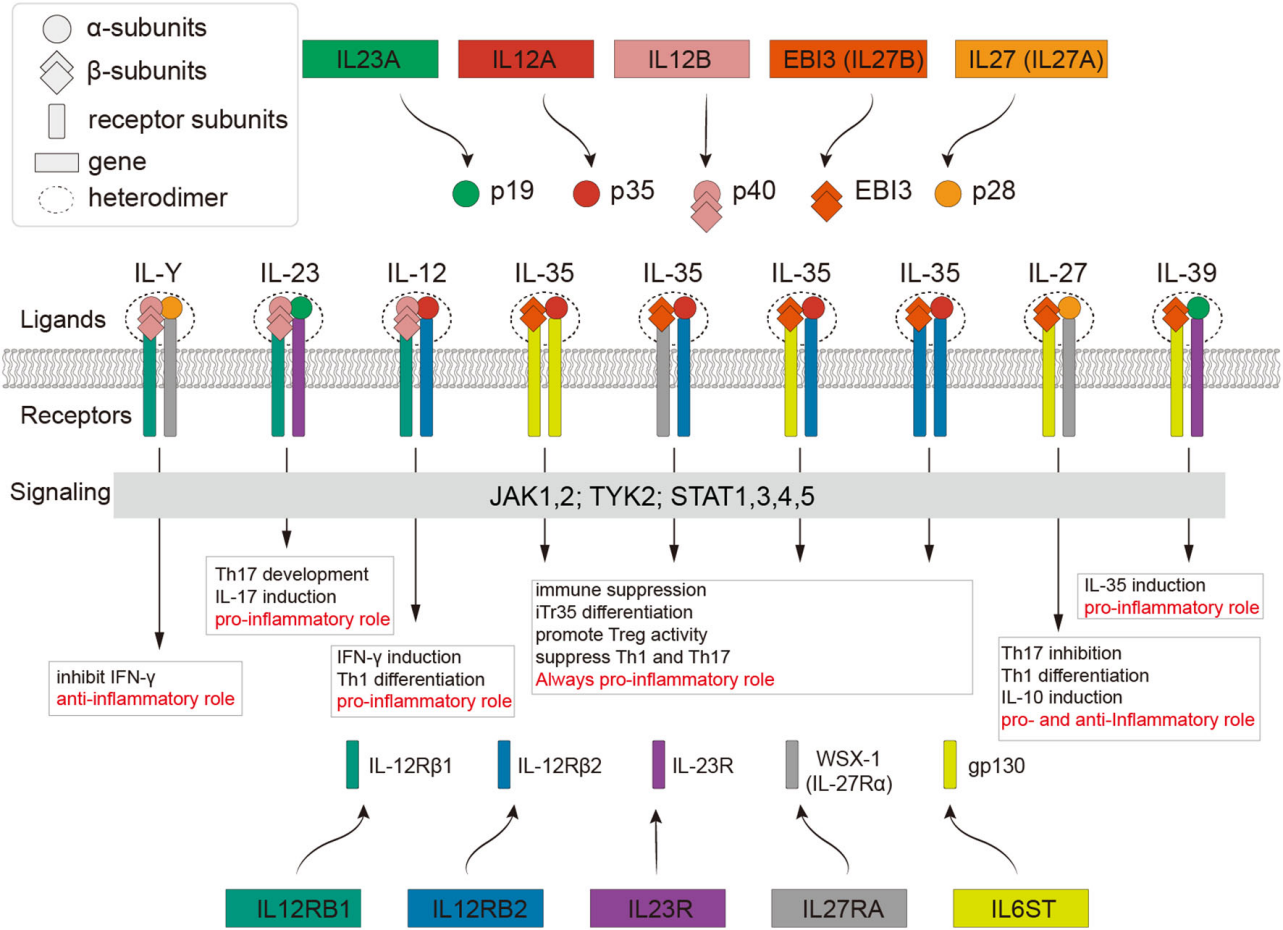
IL-12 family receptor and ligand system in humans. The same color represents genes or subunits from the same source. The text within the box indicates the specific interactions of the corresponding receptor-ligand complexes and their roles in promoting or inhibiting inflammation.

Cytokines of the IL-12 family are homodimeric or heterodimeric complexes with a four-α-helix bundle structure, similar to the IL-6 family. They are involved in autoimmune disorders and cancer progression, making them interesting therapeutic targets (1). In humans, these complexes consist of three α-subunits (p19, p28, and p35) and two β-subunits (p40 and EBI3) (**Figure 1**). The currently identified dimeric members in humans include IL-12 (p35 + p40), IL-23 (p19 + p40) (2), IL-27 (p28 + EBI3) (3, 4), IL-35 (p35 + EBI3), and the recently revealed IL-39 (p19 + EBI3) (5), as well as artificially synthesized IL-Y (p28 + p40) (6). The IL-12 family receptor dimer is composed of subunits WSX-1 (IL-27Rα), gp130, IL-23R, IL-12Rβ1, and IL-12Rβ2 (6–9). The known receptor dimers and receptor-ligand pair combinations are shown in **Figure 1**.

Cytokines in the IL-12 family, despite sharing the JAK-STAT signaling pathway, exhibit distinct biological roles. IL-12 and IL-23 are primarily pro-inflammatory cytokines that enhance the immune response. IL-12, produced mainly by antigen-presenting cells (APCs) (10), bridges innate and adaptive immunity by regulating T cells and natural killer (NK) cells, inducing IFN-γ production, and promoting Th1 differentiation (11). IL-23, also derived from APCs such as dendritic cells and macrophages, acts in both innate and adaptive immunity (2). It facilitates Th17 differentiation, activates JAK2 and TYK2, and induces STAT3/STAT4 phosphorylation (12–14). While IL-12 binds to IL-12Rβ1/IL-12Rβ2, IL-23 binds to IL-12Rβ1/IL-23R, and IL-12Rβ2 is critical for IL-12 signaling (**Figure 1**). IL-27, a heterodimer of p28 and EBI3 produced by APCs, IL-27 exhibits dual pro- and anti-inflammatory properties and promotes Th1 responses during bacterial and parasitic infections (15–18). It signals through the WSX-1/gp130 receptor complex, often associated with the IL-6 family, and synergizes with IL-12 to enhance Th1 polarization (15). In contrast, IL-23 supports Th17 development by inducing IL-17 expression and stabilizing the Th17 phenotype, although it does not directly initiate Th17 differentiation (10). Preclinical studies suggest that IL-23 promotes tumor growth and poor cancer prognosis, highlighting its potential as a therapeutic target (19). IL-35 produced by regulatory T (Treg) and B (Breg) cells has immunoregulatory functions (20–22). It signals through gp130/IL-12Rβ2, with unique signaling occurring via gp130/gp130 and IL-12Rβ2/IL-12Rβ2 homodimers (10) (**Figure 1**). IL-39, a recently discovered cytokine secreted by LPS-activated B cells, exhibits proinflammatory properties, including neutrophil proliferation and BAFF production, linking it to autoimmune diseases such as systemic lupus erythematosus (SLE) (23). EBI3, a component of IL-27, IL-35, and IL-39, features conserved cysteine residues (24). Finally, IL-Y, a synthetic cytokine that was created to investigate autoimmune illnesses and is based on fictitious IL-12 family component combinations, is not well known (6).

Comparative immunology and phylogenetics provide powerful tools for identifying universal cancer mechanisms and therapeutic targets by analyzing high-dimensional datasets and tracing cancer evolution (25, 26). However, little is known about the origin, distribution, and evolutionary features of the IL-12 receptors and ligand subunits (IL-12s and IL-12Rs) in animals. There is still much to learn about the evolutionary forces causing these variations and whether whole-genome duplication events have resulted in the amplification of IL-12s and IL-12Rs family genes (27, 28). Current therapeutic options targeting human IL-12s and IL-12Rs are limited, including ustekinumab and briakinumab targeting p40 and risankizumab, guselkumab, tildrakizumab, and mirikizumab targeting IL-23p19 (29–31). Known drugs and site-targeting ligands are even rarer, particularly for economically important animals such as fish and livestock, where research has primarily focused on recombinant IL-12 (32), anti-IL-12 antibodies (33), and receptor antagonists (33, 34). This scarcity hinders advancements in cancer therapy, agriculture, fisheries, and the livestock industry. Combining IL-12 with immune checkpoint inhibitors, such as anti-PD-1 monoclonal antibodies, significantly enhances antitumor effects (35). IL-12 can also overcome resistance to immune checkpoint blockade by providing a third signal for T-cell activation, thereby enhancing T-cell activity (36). Although checkpoint inhibitors have revolutionized cancer treatment, they face challenges related to resistance mechanisms, patient selection, and combination strategies, which parallels the evolving landscape of IL-12 cytokine-targeted therapies (37). Therefore, exploring the potential targets of IL-12s and IL-12Rs across species and elucidating their evolutionary patterns and interspecies differences could provide universal strategies for immune interventions in livestock and fisheries. In humans, this could lead to the development of precision immunotherapies, enhancing antitumor and anti-infection efficacy, improving autoimmune and chronic inflammatory conditions, and reducing the systemic toxicity of traditional drugs.

Evolutionary research has led to the concept of protein and gene families, in which individuals are classified into specific families based on their shared ancestral genes. Proteins with similar functions but no common ancestors were included in this definition (38–40). Although IL-12s and IL-12Rs do not believe they originate from a distinct gene and do not belong to the same family, they are regularly grouped as a family because of their shared structural and functional properties. In this study, sequences from more than 400 species were used in a thorough phylogenetic study of IL-12s and IL-12Rs to clarify their evolutionary trajectory and functional divergence. This comparative immunological and phylogenetic analysis sheds light on their conservation and adaptability by revealing the molecular underpinnings of the functional variations and evolutionary patterns of these cytokines.

## 2 Methods

### 2.1 Construction of the species tree

Databases such as NCBI, Ensembl, CNCB, and Macgenome were the main sources of the genomes of 518 individual animals (**Supplementary Table 1**), including GFF annotation files. These datasets were released before June 30, 2023, and contained complete annotation information for 246, 265, 6, and 1 individuals, respectively. mammalia_odb10 (https://busco-data.ezlab.org/v5/data/lineages/) was used as the database, and the GPA (https://github.com/ypchan/GPA) script was used to evaluate genome assembly quality and extract informative statistics from the Benchmarking Universal Single-Copy Orthologs (BUSCO) (41) ortholog (protein) sequences. Species with C (Complete) > 2.4%, S (Single-copy) ≥ 0.60%, D (Fragmented) ≥ 0.10%, F (Fragmented) ≥ 0.50%, and M (Missing) ≤ 97.00% were retained, and those for which the CDSs could not be extracted from the GFF files were excluded. Single-copy orthologous sequences were aligned using MAFFT v7.525 (42) with specific parameters (–genafpair –maxiterate 1000) to optimize alignment accuracy. The aligned sequences were trimmed using trimAl v1.5 (43) to remove poorly aligned positions and highly divergent regions (-gt 0.85 -cons 30). Trimmed alignments were used to construct a phylogenetic tree using IQtree v2.3.6 (44), and maximum-likelihood phylogenies were constructed using the JTT + F + R10 substitution model with 1,000 bootstrap replicates. Multiple phylogenetic trees were merged using Astral v5.7.8 (45) to generate a species tree. *Sphagnum magellanicum* was retained as the outgroup for animal phylogenetic analysis.

### 2.2 Preparation of genome and annotation files

The formats of the genome and GFF files were universally standardized. The AGAT Toolkit (46) was used to extract the longest isoform of the candidate species for genome and annotation file preprocessing. The GFF3 files that satisfied the requirements were sorted by processing the GFF files, and the script agat_convert_sp_gxf2gxf.pl was used to identify and correct errors and missing information in the GFF files. The longest isoform was preserved via the agat_sp_keep_longest_isoform.pl. All the CDS and protein sequences of the candidate species were extracted in batches via GffRead v0.12.7 (47). Finally, the CDS and protein sequences of the candidate species were combined into a single file.

### 2.3 Identification and phylogenetic analysis of the IL-12s and IL-12Rs

To identify IL-12s and IL-12Rs, we used ten query sequences of human IL-12s and IL-12Rs and their key domains (**Table 1**). A database was built by pfam_scan-1.6 (48) with default parameters, based on the combined protein file as indicated above, and members of distant species protein families were discovered using HMMER-3.3.2 (49). To identify protein sets that contained several key domains (**Table 1**), the intersection of the search results for each key domain was considered. To obtain more precise protein family information, we employed the BLASTp (50) identification method with an E-value threshold of 1e-5 to identify homologous sequences. The intersection of the Pfam and BLASTp identification results was used to determine the final identified protein set. Finally, IL-12s and IL-12Rs family trees were created using the same strategy as the species tree described above. To obtain conservative sequence information for the candidate sequence, R-script was used to extract domain and gene structure information from the Pfam search results and gene location in the GFF file.

**Table 1:** Query sequences and their domains used for the identification of IL-12s and IL-12Rs.

UniProt accession	Gene symbol (HGNC)	Protein name	Domain (Pfam)
Q8NEV9	IL27	p28*	–
Q14213	EBI3	EBI3	PF00041.26 (fn3)
P29459	IL12A	p35*	PF03039.19 (IL12)
P29460	IL12B	p40*	PF10420.14 (IL12p40_C)
Q9NPF7	IL23A	p19*	PF16649.10 (IL23)
Q5VWK5	IL23R	IL-23R	–
Q6UWB1	IL27RA	WSX-1	–
P42701	IL12RB1	IL-12Rβ1	PF00041.26
Q99665	IL12RB2	IL-12Rβ2	PF00041.26; PF06328.16 (Lep_receptor_Ig)
P40189	IL6ST	gp130*	PF00041.26; PF06328.16; PF09240.15 (IL6Ra-bind)

(*) For clarity, those proteins are designated using the consensus subunit name (–). Query sequences that did not possess Pfam domains were assigned to subfamilies exclusively based on BLASTp identification.

### 2.4 Protein family clustering and classification

All data visualization was performed using R 4.4.1, and dgfr v0.0.0.9 (51) was used to determine the ideal number of k-means clustering groups for protein sequences (min_clust = 6, max_clust = 11) and to calculate the mean/median similarity within each cluster. Ggtree v3.12 (52) and ggtreeExtra v1.14 (53) were used to enhance the aesthetics of the phylogenetic trees. Gggenes v0.5.1 (54) was used to visualize motifs, domains, and gene structures. MSA v1.36.1 (55), with the multiple sequence alignment (MSA) algorithm MUSCLE (56), was used to visualize the sequence alignment. Protein subfamilies with evident structural traits were grouped after they were found and thoroughly clustered using k-means clustering and motif, domain, and gene structure characteristics.

### 2.5 Analysis of the IL-12s and IL-12Rs Variants

Weblogo v3.7.12 (57) was used to perform SeqLogo analysis of the sequences of various groups. ESMfold (https://github.com/facebookresearch/esm), a high-precision protein structure prediction tool, was utilized for the structural prediction of regrouped protein sequences based on the pre-trained model esm2_t48_15B_UR50D (58). US-align was used for universal structure alignments of predicted structures across all redefined groups (59). Finally, UCSF ChimeraX v1.9 (60) was employed for the visualization and aesthetic appeal of the structure alignment results.

### 2.6 Synteny and origin analysis of IL-12s and IL-12Rs

We performed a site query for IL-12s, IL-12Rs, and other neighboring genes based on the Ensembl database, which includes the following species: human (*Homo sapiens*, GRCh38.p14), mouse (*Mus musculus*, GRCm39), rabbit (*Oryctolagus cuniculus*, OryCun2.0), pig (*Sus scrofa*, Sscrofa11.1), chicken (*Gallus gallus*, bGalGal1.mat.broiler.GRCg7b), and zebrafish (*Danio rerio*, GRCz11). This is because traditional synteny gene analysis is no longer sensitive enough to detect animal species with large evolutionary distances (61). Furthermore, we created a phylogenetic tree with a timescale and estimated the evolutionary period for the six species listed above, as well as *S. magellanicum* and *Caenorhabditis elegans*, based on the fossil evidence currently available in the Timetree (http://www.timetree.org/) (62).

## 3 Results

### 3.1 Identification and distribution of IL-12s and IL-12Rs in various animal Class

**Figure 2:**
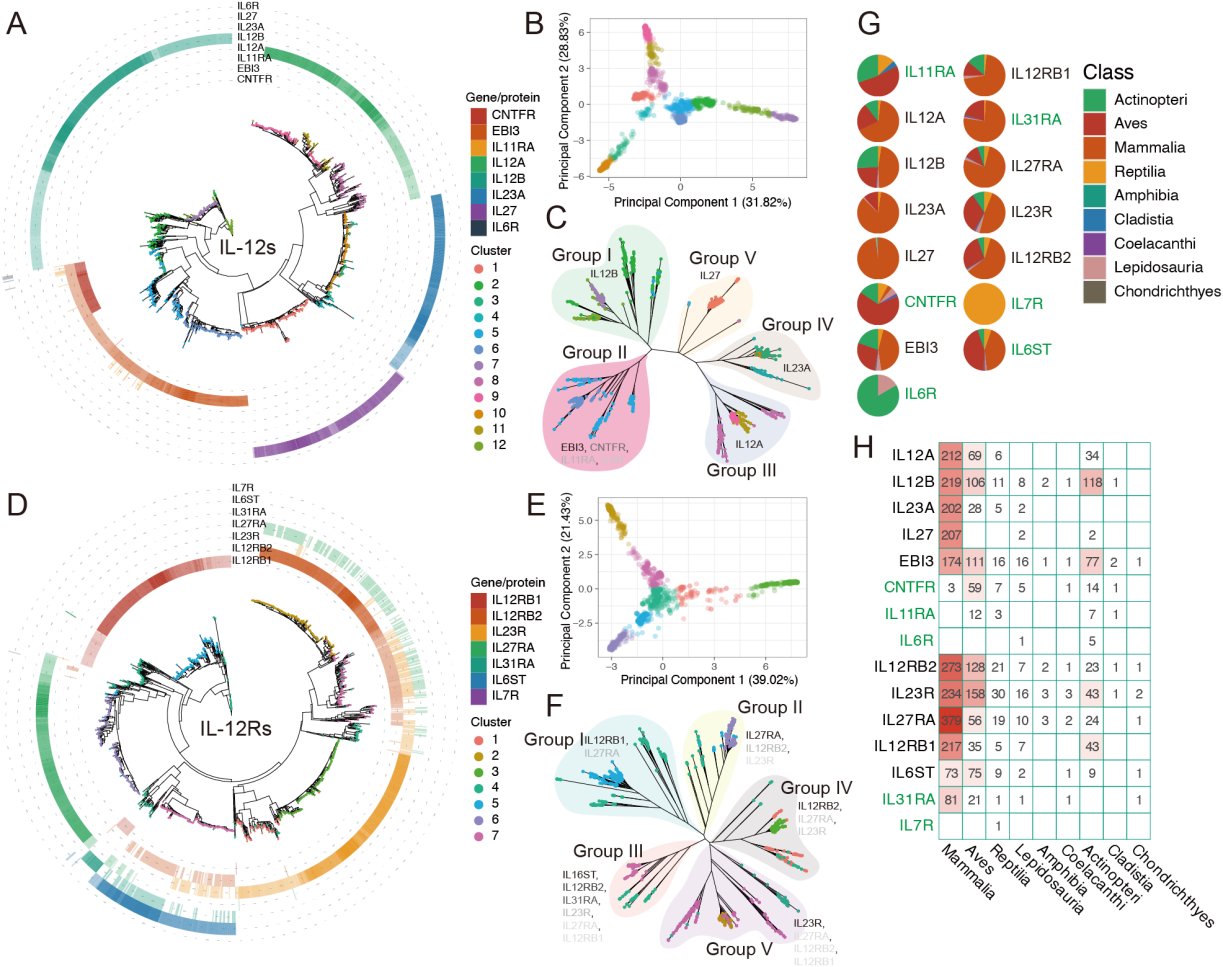
The evolutionary relationship and distribution of IL-12s and IL-12Rs in more than 400 animal species. For ease of identification, HGCN’s gene symbol naming standard was used in this figure rather than protein names (corresponding to **Figure 1**; **Table 1**). **(A**, **D)** Phylogenetic tree of IL-12s and IL-12Rs across over 400 animal species, with different colored tip points representing different clusters and different colored rings outside the tree representing homologous proteins (**Supplementary Table 1**), with the depth of color corresponding to the identity (%) of query sequences. Darker hues indicate greater identity. The relationships between IL-12s and IL-12Rs are depicted in these figures, with reference to human IL-12s and IL-12Rs, respectively. **(B**, **E)** PCA showing the total number of clusters detected in IL-12s and IL-12Rs. These clusters exhibited significant differences in the original feature space. **(C, F)** Phylogenetic tree and distribution patterns of IL-12s and IL-12Rs. The gene symbol font color depth indicates the broad distribution of the main homologous protein types in the groups, which are represented by gene symbols that match human homologous genes. **(G**, **H)** Distribution of IL-12s and IL-12Rs at the Class level. The distribution of genes in green exclusively represents the distribution of homologous proteins with IL-12s or IL-12Rs; it does not represent the distribution of all homologous proteins in the species.

We constructed a phylogenetic tree spanning approximately 1,290 million years, encompassing 491 individual animals (https://github.com/ka3533/Family_IL-12s_Rs), including 480 animal species and an outgroup of *S. magellanicum*. The selected species ranged from higher to lower taxa, including Mammalia, Aves, Actinopteri, Lepidosauria, and Reptilia. After obtaining the intersection of the protein sets identified via the Pfam and BLASTp methods, we acquired a phylogenetic tree of 1,634 IL-12s-like protein sequences from 410 individuals (405 species) (**Figures 2A, C**) and a phylogenetic tree of 1,396 IL-12Rs-like protein sequences from 396 individuals (391 species) (**Figures 2D, F**).

**Figure 3:**
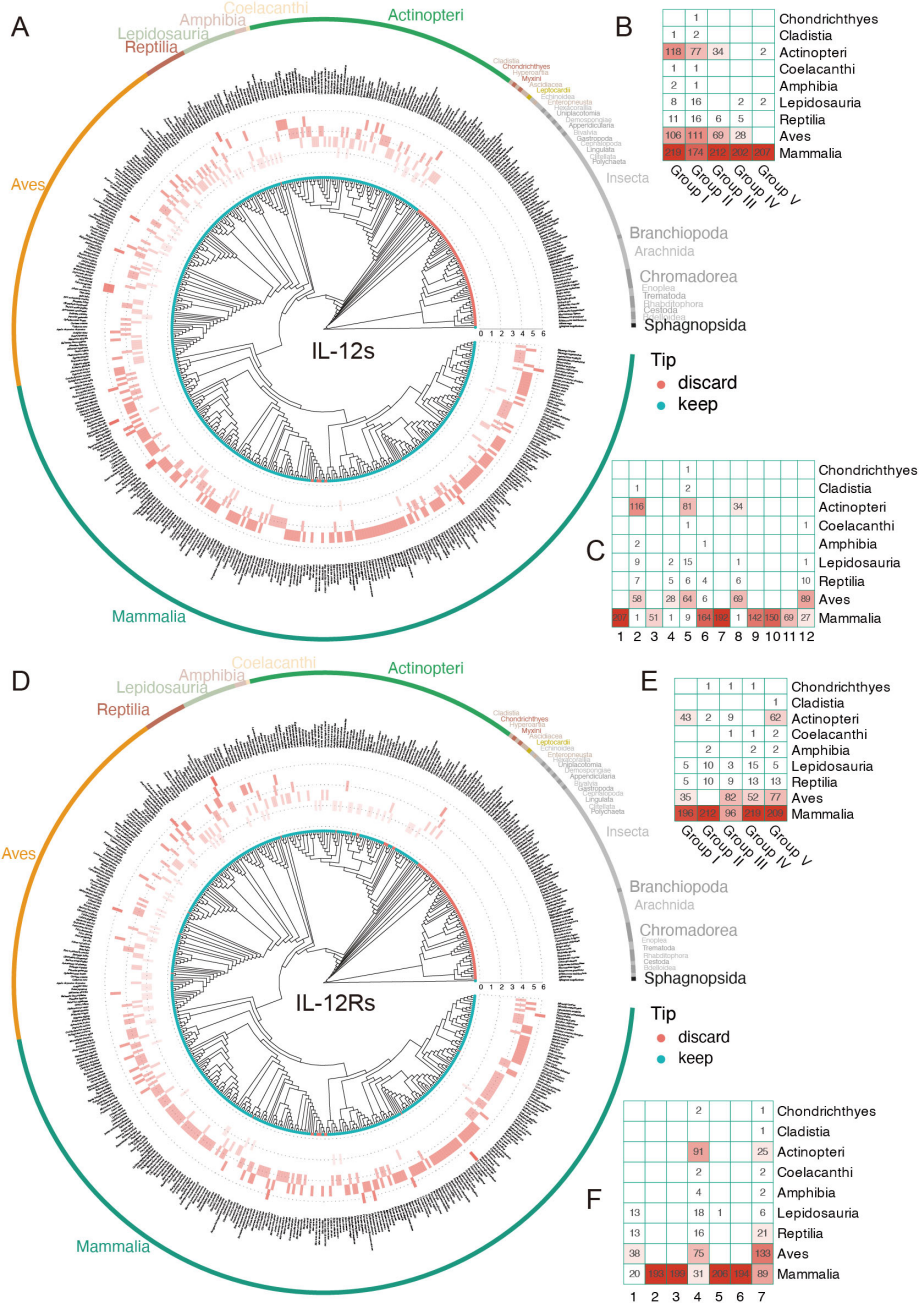
Distribution of IL-12s and IL-12Rs gene counts across over 400 animal species. **(A**, **D)** Distribution of the IL-12s gene in 410 individuals (405 species), and the distribution of the IL-12Rs gene in 396 individuals (391 species). The tip points in cyan represent species individuals retained following BUSCO assessment, whereas the red points indicate the species that were filtered out; however, this does not mean that the filtered species were absent, but rather that the overall data did not meet the standards. **(B, E)** Gene counts of IL-12s and IL-12Rs homologs at the Class level after grouping. **(C**, **F)** The distribution heatmap of PCA clustering of IL-12s and IL-12Rs at the Class level before grouping; the abscissa represents the cluster number, corresponding to **Figure 2**.

To obtain a clear classification, we also performed PCA clustering on the IL-12s, combining their homology with human IL-12s, homologous protein categories, and unrooted trees to categorize them into different Group I-V (**Figure 2C**). Apart from EBI3 homologs, other IL-12s did not show significant overlap in the phylogenetic tree, with each forming an independent cluster. However, EBI3 homologs are highly similar to CNTFR (**Figure 2A**), and a few EBI3 homologs also exhibit homology with IL-11RA and IL-6R. Since these proteins seem to be very ancient (**Figure 3C**: cluster 5), mostly found in a few ancient lineages in Actinopteri, Aves, and Reptilia, and nearly nonexistent in Mammalia, they may be ancient forms of EBI3 homologs (**Figure 2H**). p28 homologs are predominantly found in Mammalia (98.1%), with an identity higher than 47.03%, and most of them have an identity above 70%. In Lepidosauria and Actinopteri, only four extremely low-identity (27.03%-27.36%) proteins were detected in *Denticeps clupeoides*, *Pantherophis guttatus*, and *Python bivittatus*. In addition, all other p28 homologs belonged to cluster 1 (**Figures 2A, C**), indicating low sequence polymorphism and evolutionary conservation, divided into Group V. In conclusion, it seems that p28 homologs are specific to Mammalia (**Figure 2H**). p19 homologs are widely distributed in Mammalia (85.23%) and Aves (11.81%), with a small presence in Reptilia (2.11%) and Lepidosauria (0.84%), found only in clusters 3, 4, and 10, categorized as Group IV. The same phenomenon occurs with p35 homologs as well. These proteins were broadly distributed in Mammalia (66.04%), Aves (21.5%), Actinopteri (10.59%), and Reptilia (1.87%) and were found only in clusters 8, 9, and 11, which were categorized into Group III. This showed that teleosts might not have p19 homologs, which gradually became enriched in the later stages of avian evolution. p35 homologs, on the other hand, are the opposite (**Figure 2H**). p40 and EBI3 homologs are the most widely distributed proteins and are present in most animal categories. Unlike other ligand subunits, the β-subunits p40 and EBI3 homologs are abundantly present in Actinopteri (**Figures 2G, H**), which is the oldest form of IL-12s.

For IL-12Rs, we employed the same clustering and grouping strategies. gp130 homologs are widely present in Aves (44.12%) and Mammalia (42.94%), being the most extensively distributed IL-12s in Aves (28.91-61.45%), which is found only in clusters 4 and 7 of Group III, with its identity gradually decreasing from Mammalia to Chondrichthyes. This suggests that despite the paucity of available Aves genomic data, it is undeniable that gp130 homologs are crucial to the immune system of Aves and that their functions may be too dissimilar from those of Mammalia. Other IL-12s are most extensively distributed in Mammalia; however, IL-12Rβ2 homologs are far more abundant in Aves (59.74%) than in IL-12Rβ1(**Figures 2G, H**). The distributions of IL-23R and WSX-1 homologs were quite similar; WSX-1 homologs were more concentrated in Mammalia (76.72%), being the most abundant IL-12Rs gene in Mammalia, followed by IL-12Rβ1 homologs (70.68%). IL-12Rβ1 homologs can be linked back to the Actinopteri period, but the homologous genes of p28, IL-23R, and IL-12Rβ2 are widespread and most likely date back to the Chornichthyes period with *Callorhinchus milii* (**Table 2**).

**Table 2:** The oldest traceable IL-12s and IL-12Rs genes/proteins across over 400 animal species.

Longest isoform ID	Identity	Homologue gene	Homologue protein	Ancient animal	Class	Group
ENSECRT00000020045	29.85%	IL12B	p40	*Erpetoichthys calabaricus*	Cladistia	Group I
ENSCMIT00000045986	39.39%	EBI3	EBI3	*Callorhinchus milii*	Chondrichthyes	Group II
ENSECRT00000011998	31.79%	IL11RA	IL11RA	*Erpetoichthys calabaricus*	Cladistia	Group II
ENSECRT00000011998	60.26%	CNTFR	CNTFR	*Erpetoichthys calabaricus*	Cladistia	Group II
ENSDART00000145103	29.58%	IL6R	IL6R	*Danio rerio*	Leptocardii	Group II
ENSLOCT00000004429	29.70%	IL12A	p35	*Lepisosteus oculatus*	Actinopteri	Group III
XM_034413038.1	35.25%	IL23A	p19	*Pantherophis guttatus*	Lepidosauria	Group IV
ENSDCDT00000036795	27.27%	IL27	p28	*Denticeps clupeoides*	Actinopteri	Group V
ENSDCDT00000070609	27.27%	IL27	p28	*Denticeps clupeoides*	Actinopteri	Group V
ENSLOCT00000001699	25.56%	IL12RB1	IL-12Rβ1	*Lepisosteus oculatus*	Actinopteri	Group I
ENSCMIT00000011517	26.67%	IL27RA	WSX-1	*Callorhinchus milii*	Chondrichthyes	Group II
ENSCMIT00000011517	24.34%	IL23R	IL-23R	*Callorhinchus milii*	Chondrichthyes	Group II
XM_037888031.2	26.48%	IL7R	IL7R	*Chelonia mydas*	Reptilia	Group II
ENSCMIT00000047827	32.26%	IL31RA	IL31RA	*Callorhinchus milii*	Chondrichthyes	Group III
ENSCMIT00000047827	24.36%	IL12RB2	IL-12Rβ2	*Callorhinchus milii*	Chondrichthyes	Group III
ENSCMIT00000047827	39.87%	IL6ST	gp130	*Callorhinchus milii*	Chondrichthyes	Group III
ENSCMIT00000037583	33.33%	IL23R	IL-23R	*Callorhinchus milii*	Chondrichthyes	Group IV

Among the oldest ancient species (traceable only in this study), the identified IL-12 and IL-12 receptor (IL-12R) sequences showed relatively low identity with their query sequences (**Table 2**). However, these ancestral proteins retained the canonical structural and sequence features that are characteristic of IL-12s and IL-12Rs signaling components (see below). Notably, these species, particularly *C. milii* and *E. calabaricus*, represent valuable models for reconstructing the evolution of early immune systems. In conclusion, IL-12Rs and IL-12s (apart from p28-like proteins) have comparatively ancient origins that predate the emergence of Chornichthyes. The ancient innate immune system, composed of these receptors and ligands, may have gradually expanded its range of function during the emergence of Aves.

### 3.2 Evolutionary patterns of IL-12s and IL-12Rs across several groups

To explore the evolutionary patterns of genes encoding IL-12s and IL-12Rs, we mapped the distribution of quantities on the species tree, which revealed that both IL-12s and IL-12Rs underwent minor quantitative changes from ancient animals to today’s immune system-developed mammals (**Figures 3A, D**). This is particularly prominent in Mammalia, where the gene number of IL-12s and IL-12Rs mostly exceeds five, with a few species reaching seven, making them the most abundant Class in the IL-12s and IL-12Rs (**Figures 3A, D**). Unlike Mammalia, Aves, with a slightly greater number of IL-12Rs than IL-12s, a few Aves species can have as few as two IL-12s or IL-12Rs genes, a situation that is very rare in Mammalia. Most interestingly, in Actinopteri, which is mainly composed of teleosts, the numbers of IL-12s and IL-12Rs showed the opposite trend, with the ligand number being far greater than the receptor number. Most species had more than three IL-12s genes, whereas the receptor genes were mostly maintained at two (**Figure 3D**). The primary cause of these phenomena is the duplication of the IL12B and EBI3 genes, as well as their long-term retention following duplication events.

The number of genes encoding receptors and ligands in certain species, as well as the distribution of IL-12s and IL-12Rs across species, both reflect their ancient origins. Even among ancient teleosts, as shown in **Figures 3A, D**, some species had more than four receptor genes, especially when considering ligand genes, which are far more common than receptor genes. This further supports the idea that the IL-12 immune system is an ancient immune system. However, it was not until terrestrial creatures appeared that receptor diversification took place, peaking in Mammalia.

### 3.3 Conserved characteristics of IL-12s and IL-12Rs across several groups

The IL-12 family is divided into five groups: Group I contains an IL12p40_C domain (sometimes preceded by Ig-like domains), Group II has fn3 (occasionally with Ig-like domains), Group III and IV consists of single IL12 or IL23 domains, while Group V includes only five sequences with CNTF, the rest being p28-like. Consequently, the IL-27 family lacks a seed alignment with broad-spectrum characteristics that can effectively identify p28-like proteins. Among IL-12Rs, most contain fn3 domains—Group I has a single fn3, Group II has 1–2 fn3 at the C-terminus, Group III features Lep_receptor_Ig followed by fn3 (sometimes with IL6Ra-bind or EpoR_lig-bind), Group IV primarily has IL6Ra-bind (rarely followed by fn3), and Group V mostly contains Lep_receptor_Ig with 2 fn3 (some with IL6Ra-bind) (Supplementary **Table 2**). The prevalence of fn3 (except in Group IV) highlights its potential in biomedical applications owing to its role in cell adhesion and migration. In conclusion, animal IL-12Rs do not always have Ig-like and fn3 domains as is generally assumed. Moreover, the number of sequences possessing this domain in Group II and IV is extremely limited, indicating that in current research, the subfamilies corresponding to WSX-1 and IL-12Rβ2 have not been fully characterized at the sequence level. In addition, some animal IL-12s still had domains similar to those of Ig-like, such as Group I (**Supplementary Table 2**).

**Figure 4:**
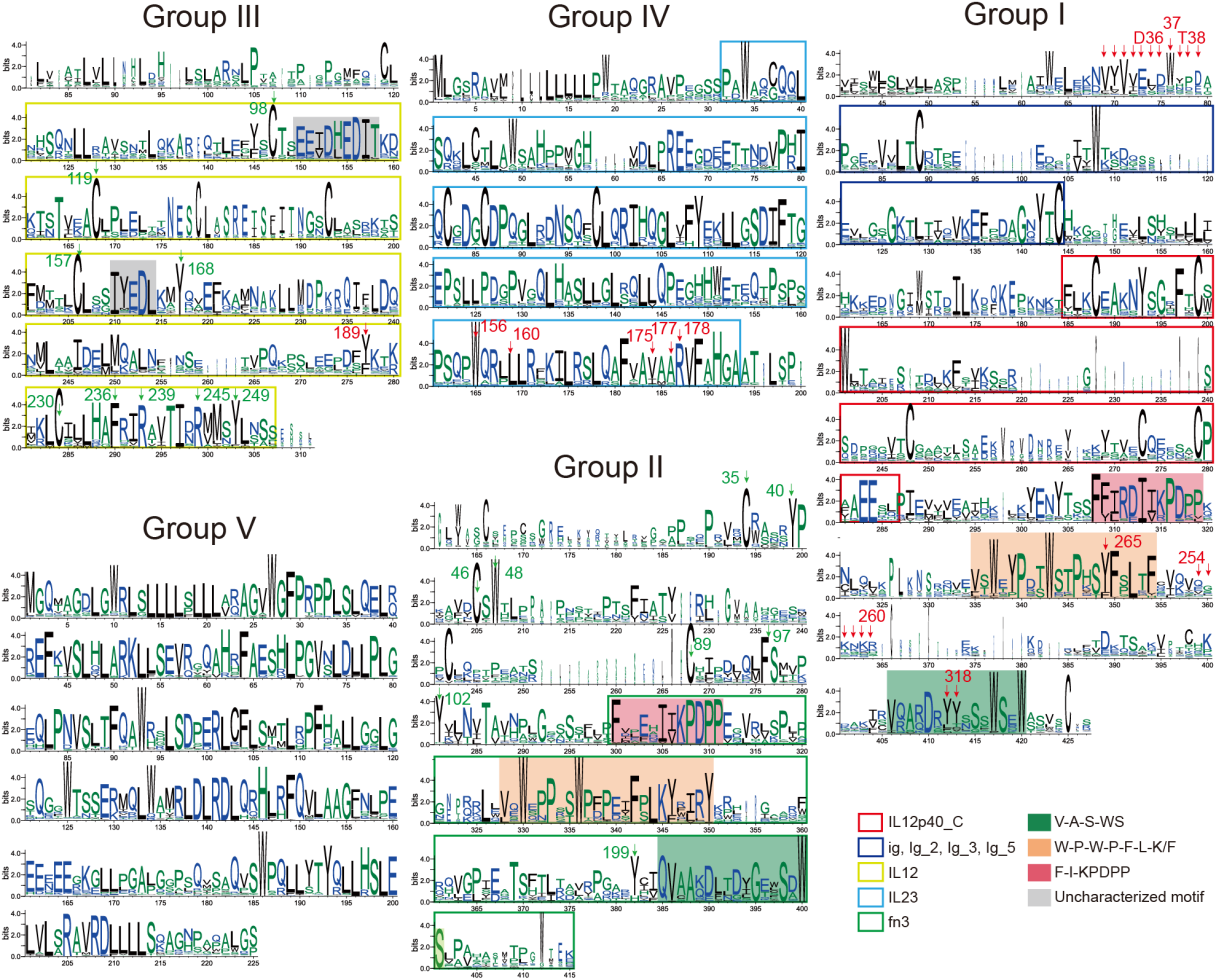
Conserved features of IL-12s during animal evolution. SeqLogo plots of the five subgroups of IL-12s (trimmed), explaining the conserved sequence features of the five groups of IL-12s in 405 species of animals that have revealed genomes to date, and the features of the domains (Pfam) that can be detected are indicated by intervals covered by the colored blocks. The key residues, that have been demonstrated, are indicated by arrows; the key residue sites speculated in this study are indicated by green arrows (excluding the highly conserved Group IV and V). These positions were identified based on the sequence features surrounding the key residues. The figures reveal potential therapeutic targets within the various groupings of 405 animal IL-12s, particularly focusing on the residues within the loop regions and domains that significantly affect protein structure.

**Figure 5:**
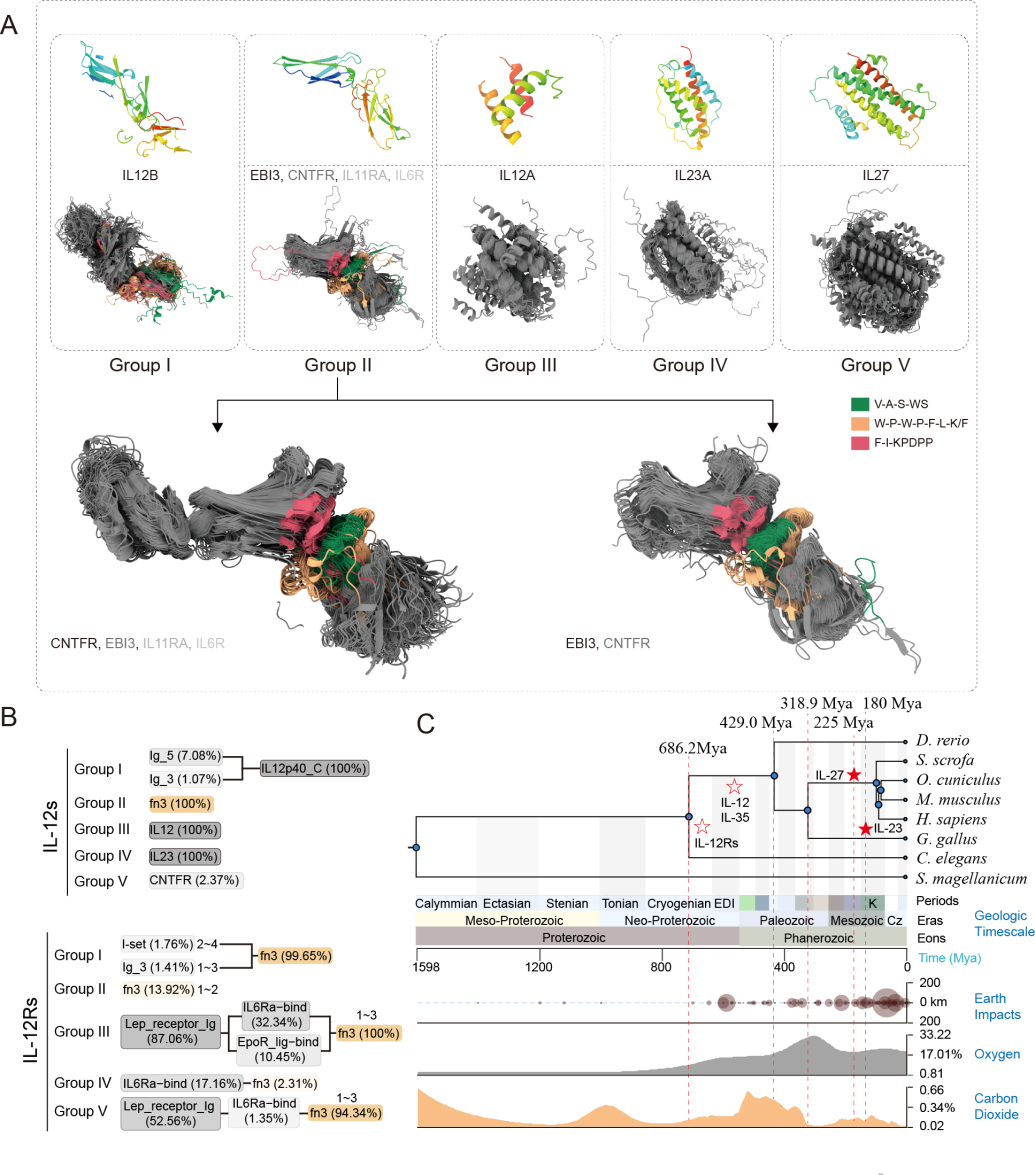
The evolutionary trajectory of IL-12s and their ultra-conserved spatial structures were maintained during evolution. **(A)** Spatial structure comparison results of the proteins in each subgroup of IL-12s. The top is the structure of the unique proteins of the subgroup, and the bottom is the spatial structure comparison result of US-align. The protein type is represented by the gene symbol that corresponds to the longest transcript. The non-conserved spatial structure region was set to 100% transparency. SeqLogo is shown in **Figure 4**. The gene symbol font color depth indicates the broad distribution of the main homologous protein types in the groups, which are represented by gene symbols that match human homologous genes. The general characteristics of the β-subunits of animal IL-12s are shown in this figure. The α-subunits, on the other hand, only preserve their conserved three-dimensional structure; the unified sequence features of these three groups are very weak. **(B)** Redefined domain characteristics were preserved during the evolution of IL-12s and IL-12Rs across different subgroups, with domains accounting for less than 1% of the total proportion filtered out. **(C)** The evolutionary trajectory of IL-12 family receptor-ligand complexes, where solid stars indicate precisely estimated time points of receptor-ligand complex emergence and hollow stars represent roughly estimated time points.

Protein sequences in each group were subjected to MSA, trimming, and SeqLogo analysis, which revealed that Group I and II sequentially contain three highly conserved motif structures (“F-I-KPDPP”, “W-P-W-P-F-L-K/F”, “V-A-S-WS”) at the C-terminal end, with “-” representing intervals of one or more amino acids. We found that these three signatures (**Figures 4**, **5A**) were distinct characteristic sequences within the fn3 domain. The key amino acids, Y265 and Y318 in earlier research (63), which are crucial parts of the β subunits in animal IL-12s, are covered by these three conserved structures.

In contrast, owing to the limited characterization of existing Pfam seed files, we did not detect fn3 in the interval of Group I, but we found that the C-terminus after IL12p40 in Group I is also an fn3-like domain (**Figures 4**, **5B**). Therefore, our analysis using SeqLogo of homologous proteins from over 400 animal species provides a greater representation, and we located the confirmed key sites based on sequence features (**Figure 4**). Despite the low overall identity between Group I and II, both groups share similar motif structures in the C-terminal portion of their proteins (**Figures 4**, **5A**). The gene coding for the p35 homologous protein is found in Group III, which is the only group not enriched in tryptophan (W), but it has an ultra-conserved cysteine (C), similar to Group I, II, and IV. Few reports exist regarding conserved amino acids and motifs. Multiple-species sequence alignment of p35 homologous proteins revealed that the conserved regions of Group III were concentrated in the four α-helical regions of p35 (**Figures 4**, **5A**). In this structure, only a few key residues have been studied and validated, such as Y189 (63). Additionally, we have identified several conserved residues and motifs across species that may play roles in maintaining protein structure and function, including C98, C119, C157, C230, “EETDHEDIT”, “IYEDL”, and the highly conserved C-terminal region (**Figure 4**: Group III). The non-conserved regions exhibited species-specific characteristics (except for the N-terminus), with a large number of similar species-specific peptide segments. Group IV also exhibits ultra-conserved structural features, with its non-conserved region mainly concentrated at the N-terminus, which is in direct contrast to the non-conserved region of Group V. This group exhibits a long-ultra-conserved sequence architecture in MSA, even before trimming, demonstrating remarkably low polymorphism. This evolutionary pattern suggests functional uniformity, consistent with its characterization as a mammalian-specific ligand subunit with singular biological activity. Notably, numerous critical C-terminal residues have been identified, including W156, L160, V175, A177, and R178 (63). Exceptions are observed in certain carnivoran species (*Neogale vison*, *Lontra canadensis*, *Lutra lutra*, *Mustela erminea*, and *Mustela putorius*) and primate lineages (*Macaca thibetana* and *Symphalangus syndactylus*). These taxa encode typical p19-like proteins containing substantial insertions, resulting in pronounced species-specific structural divergence from the canonical protein architecture. Group V, which represents the p28 protein family, is the most conserved among all IL-12 family members (**Figures 2A, C**) and possesses the richest tryptophan amino acids, which are almost exclusively distributed among Mammalia (**Figure 2H**). This implies that the p28-like protein, a component of the IL-12 family of proteins that only manifests during the mammalian phase, maybe the most structurally and functionally stable in Mammalia. However, current research on the critical functional sites of this family remains limited. This indicates that the four-helix structure has been preserved throughout the long evolutionary process of animal p19, p28, and p35 homologous genes, and their three-dimensional structure is essentially the same, even if their sequences vary significantly. This implies that the three-dimensional structure of the alpha subunit is essential for the IL-12Rs recognition mechanism.

All animal IL-12Rs-like protein sequences exhibited a distinct and recognizable “WSXWS” motif feature (**Supplementary Figure 1**), with a significant number of conserved cysteines at the N-terminus that may help maintain the receptor’s three-dimensional structure by forming disulfide bonds. In contrast, groups II and III possess highly conserved overall sequence structures, particularly at the C-terminus, whereas the other groups demonstrate conservation only in specific regions and display species-specific characteristics. Currently, little information is available on the key IL-12Rs residues. The identification of key regions is severely limited, although we disclosed their conserved sequence characteristics (**Supplementary Figure 1**). Consequently, additional experimental data are required to identify their primary target locations. However, the conserved sequence features that we have identified can serve as a useful guide for IL-12Rs target screening in a variety of species.

### 3.4 Key targets of IL-12s

As illustrated in **Figure 4**, most of the conserved sites identified in homologous proteins across various groups play crucial roles in drug development and are located predominantly within key structural domains in more than 400 animal species (**Figures 4**, **5**). Notable examples include the C-terminal Y265 and Y318 (63) and the amino acid cluster 254-260 (64) in Group I, as well as the N-terminal cluster of amino acids 30-39 (65); the Y189 in the IL12 domain of Group III (63); and the W156, L160 (63), and V175, A177, R178 (63) in the IL23 domain of Group IV (**Figure 4**). However, reports on drug targets or key amino acids corresponding to Groups II, III, and V of IL-12s are limited, and IL-12Rs are even more so (**Supplementary Figure 1**). Through cross-species MSA, we have annotated potential key residues and motifs that may influence the structure and function of IL-12s, particularly three signature regions within the fn3 domain (**Figures 4**, **5A**). These regions may serve as critical amino acid targets for broad-spectrum drug development, especially considering the evolutionarily conserved residues across Group I, II, and III species. However, regarding IL-12Rs’ key sites, which are limited by current sparse research data, exceptionally long protein sequences (**Supplementary Figure 1**), and complex spatial structures, this study refrains from further elaboration.

### 3.5 Origin of IL-12s and IL-12Rs

Our findings suggest that the EBI3, IL12B, CNTFR, IL11RA, and IL6R genes may share a common evolutionary origin (**Figure 2C**). However, current evidence remains insufficient to support a common evolutionary origin, as the synteny analysis results do not provide conclusive support for this hypothesis (**Supplementary Figure 2A**). Its genesis might be extremely ancient, and we hypothesize that it might have existed before the mollusk era (**Figure 5C**). Among the five groups of IL-12s, Group I, II, and III are widely distributed in Actinopteri, Aves, and Mammalia, especially in teleosts, with clear third or fourth whole-genome duplication (WGD) traces (**Figure 3B**). The Group I and V subgroups of receptors likewise exhibit this situation (**Figure 3E**). However, both CNTFR and IL11RA are found on 9p13.3, and their gene positions are adjacent in teleosts and Aves, with no other genes in between; only in the evolution to Mammalia do they gradually become separated by other genes, such as GALT, SIGMAR1, ARID3C, DCTN3, and RPP25L. The linear arrangement order of genes in this region is highly consistent across species, suggesting that CNTFR and IL11RA may have a common ancestor and may have been the product of an ancestral gene duplication event. According to synteny analysis, which revealed that IL12RB2 is nearly always adjacent to the IL23R from zebrafish to humans and that the majority of proteins similar to IL-23R are also similar to IL-12Rβ2 (**Figure 2D**), these pieces of evidence suggest that the homologs of IL27RA, IL23R, and IL12RB2 may be among the older three types of IL-12Rs. IL23R and IL12RB2 may have come from an ancient gene duplication event. Comparative analysis suggests that IL-12Rs may have originated earlier than IL-12s, as genes encoding various IL-12Rs subtypes are widely distributed across nearly all animal species. Notably, most of these receptors contain evolutionarily ancient domains such as fn3 (66), implying that the fn3 domain likely emerged in its primitive form during early vertebrate evolution. Supporting this hypothesis, our rough BLAST searches identified only ancestral fn3 and Ig-like genes in nematodes, which may represent the prototypical precursors of modern cytokine receptors.

Based on available fossil evidence, we reconstructed a time-calibrated phylogenetic tree of model species. Combined with synteny analysis, our results demonstrate that the genes encoding IL-12 ligands and receptors have maintained remarkable evolutionary conservation throughout vertebrate history. Even in ancient species, their surrounding genes are comparable to that of higher animals (**Supplementary Figure 2**). Aside from the p28 and p19 homologous genes, other IL-12s and IL-12Rs may have already appeared as early as the distant Nematoda period (~686.2 million years ago, Mya) and can at least be traced back to the Chondrichthyes period (~429.0 Mya). The origin of IL-12Rs can even be traced back to earlier, and we speculate that their embryonic form occurred during the mollusk period, which is ~514.0 Mya (67). These findings are in line with the distribution of IL-12s and IL-12Rs genes across species. The p28 ancestor genes, on the other hand, emerged in significant quantities during the mammalian epoch (~225 Mya), however, their actual origin may have been as early as the reptile period (**Figure 5C**). In the IL-12 family, the protein subunits of Group I to III (p40, EBI3, p35) are relatively ancient, as they are widely distributed among fish species. In contrast, the newly evolved ligand subunits of Group IV to V became abundant only after the emergence of avian species—for instance, p19. Notably, p28 is unique as it is exclusively found in mammals (**Figures 3B, C**: cluster 1). Among the heterodimeric IL-12 family protein ligands, IL-23 and IL-39 gradually increased in abundance after the rise of birds, whereas IL-27 represents a mammalian-specific immune system innovation. Conversely, IL-12 and IL-35 have much older evolutionary origins, likely predating even the Chondrichthyes period (**Figure 3C**: clusters 2, 5, 8). Regarding IL-12Rs, nearly all subtypes exhibit ancient origins, possibly predating IL-12s themselves, given their near-universal presence across animal species (**Figure 3E**). However, extensive duplication occurred exclusively in mammals, as seen in clusters 2, 3, 5, and 6 (**Figures 2F**, **3F**). Since IL-12Rs are evolutionarily ancient, we can infer the origins of the IL-12 receptor-ligand system based on the emergence of IL-12s—particularly for the newly discovered IL-35 and IL-39. The formation and initial function of the IL-27/WSX-1/gp130 complex coincides with the origin of mammals (~225 Mya). In contrast, the IL-23/IL-12Rβ1/IL-23R complex predates mammals, originating around the early evolution of birds (~180 Mya). The assembly of IL-12/IL-12Rβ1/IL-12Rβ2 and IL-35-type ligand-receptor complexes dates back to approximately ~686.2 Mya.

## 4 Discussion

### 4.1 Evolutionary insights and ancient origins

Higher animals have an advantage over other creatures in part because of the complexity of their IL-12 immune systems. Other cytokine families may likewise exhibit a similar pattern. Class I cytokine receptors diverged mostly between the urochordate-vertebrate split (~794 Mya) and the ray-/lobe-finned fish divergence (~476 Mya), with little further diversification (68). Then vertebrate animals evolved from invertebrates some 500 Mya through two rounds of WGD (2R) (69, 70). Chondrichthyes separated from a common ancestor with other vertebrates roughly 450 Mya (71, 72). Since the advent of jawed vertebrates, the tripartite subdivision of lymphocytes bearing altered receptors into B cells and T cells has been retained. This step is considered the founding stage of the animal adaptive immune system (73, 74). There was a subsequent teleost-specific WGD (3R, ~300 Mya), with numerous distinct lineages having additional WGD events, such as salmonids (4R, ~95 Mya) and carp (4R, 5.6-11.3 Mya) (69, 70). Our research has indicated that the number of IL-12s and IL-12Rs genes does not increase exponentially due to WGD (**Figure 3**). Instead, gradual and subtle changes in gene numbers have occurred throughout evolution. Despite these minor numerical changes, their impact on the evolutionary history of animals has been significant. Apart from mammalian-specific p28 homologs and p19, other IL-12s and IL-12Rs likely emerged as early as the Nematoda period (~686.2 Mya) and can be traced back at least to the Chondrichthyes period (~429.0 Mya) (**Figure 5C**). The genomic arrangement of these genes and their neighboring genes is highly conserved, though some genes exhibit clear duplication events, such as IL11RA and CNTFR, IL31RA and IL6ST, and IL23R and IL12RB2 (**Supplementary Figures 2A, B**).

**Figure 6:**
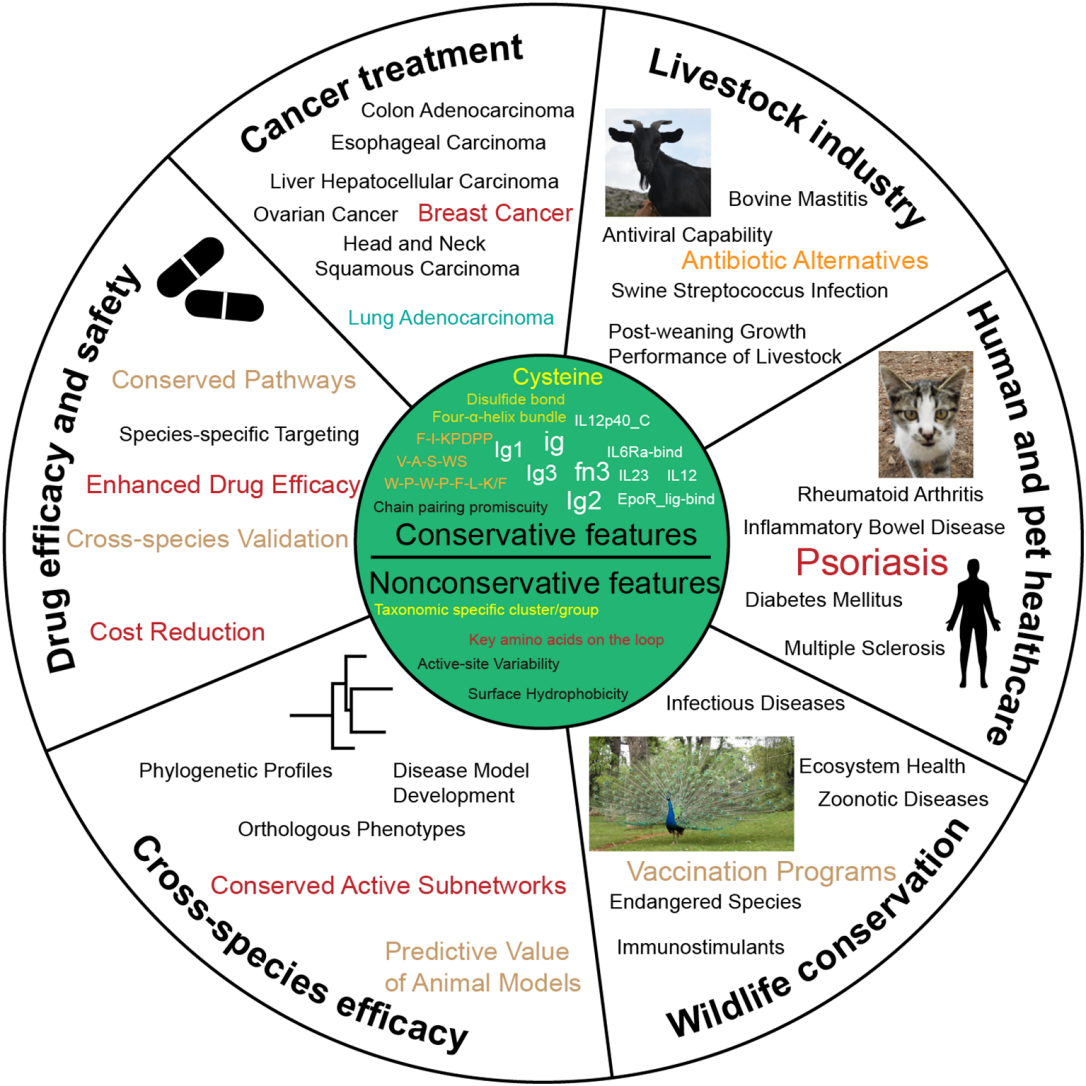
Potential uses for the conservative and nonconservative features of IL-12s and IL-12Rs. This figure offers recommendations for cross-species IL-12s and IL-12Rs application strategies.

From an evolutionary perspective, the origin and functional diversification of these molecules reflect the progression of the immune system from simplicity to complexity. By comparing IL-12s and IL-12Rs among different species, we have identified evolutionarily conserved residues, motifs, and domains that likely play pivotal roles in immune regulation. These conserved features may serve as potential therapeutic targets for developing precision immunotherapies applicable across multiple species. Furthermore, understanding species-specific structural and functional variations could facilitate the design of tailored immunomodulators for specific animals, enhancing health and productivity in livestock while offering novel strategies for agriculture and aquaculture (**Figure 6**). Thus, multispecies studies not only illuminate the evolutionary trajectory of the immune system but also provide critical insights for cross-species drug development and disease control.

### 4.2 Significance of IL-12 family in immune regulation

IL-12s and IL-12Rs are associated with a variety of human and other animal diseases, such as psoriasis (75), Crohn’s disease (CD) (76), rheumatoid arthritis (RA) (77), diabetic retinopathy (DR) (78), pancreatic cancer (PC) (79), central nervous system (CNS) autoimmune diseases (80), and tumor immunotherapy (81), among many other immune-related disorders. However, current effective treatments for these diseases remain limited, and challenges extend beyond receptor-ligand affinity, receptor-ligand inhibitors, and targeted drug screening and validation. Exploring potential therapeutic targets based on the conserved or nonconserved features of IL-12s and IL-12Rs across various animal groups may facilitate the development of treatments for these diseases.

For instance, IL-23p19 inhibitors such as risankizumab, guselkumab, tildrakizumab, and mirikizumab demonstrate a favorable risk-benefit ratio in patients with moderate-to-severe psoriasis (75) and are more effective than ustekinumab (which is widely approved in multiple countries for CD treatment) (76, 82, 83). These inhibitors significantly improve psoriasis symptoms, and selective targeting of IL-23p19 may help avoid adverse events associated with biologics employing other mechanisms, exhibiting good safety profiles (75, 84, 85). IL-23p19 inhibitors also hold potential for development in inflammatory bowel disease (IBD) treatment (86). Currently, IL-23 or IL-23 receptor targeting has been proposed as a potential therapeutic strategy for RA, but further clinical research and target validation are needed to confirm efficacy and safety (77, 87). IL-35 provides critical insights into the application of autoregulatory B and T cells in treating human diseases and shows potential clinical value in central nervous system autoimmune disease therapy (80). However, the current understanding of the immunobiology of IL-35 and its subunits remains limited, necessitating further research to clarify its specific roles and applications in disease treatment. IL-27 can stimulate cytotoxic T-cell activity and may play a role in tumor immunotherapy (81). Currently, therapeutic strategies targeting p28 are still in the research phase. IL-39 plays a key role in the pathogenesis of chronic graft-versus-host disease (cGVHD) and may serve as a potential therapeutic target for cGVHD prevention. It also holds diagnostic potential in RA (88), ankylosing spondylitis (AS) (89), type 2 diabetes mellitus (T2DM) (90), breast cancer (BC) (91), and periodontitis (92). Additionally, IL-39 may function as an inflammatory mediator in active IBD and could emerge as a novel treatment option (93). EBI3 may contribute to the anti-tumor effects of IL-27, though further research and clinical validation are required to determine its specific applications in tumor immunotherapy (81).

Research on IL-12Rs-related diseases is notably scarce compared to that on ligands. For example, in the MRL/lpr mouse model of systemic lupus erythematosus (SLE), overexpression of WSX-1 significantly ameliorated autoimmune phenotypes (94). This finding suggests that enhancing the IL-27 signaling pathway may be a potential strategy for treating autoimmune diseases like SLE and could also improve immune responses in chronic viral infections (95). gp130 is one of the receptors closely associated with tumors (96). Studies indicate that the gp130-mediated signaling network plays a crucial role in the progression of various cancers. Its inhibitors effectively suppress tumor cell viability, migration, and promote apoptosis, while also inhibiting tumor growth in xenograft models (97). This discovery provides a new direction for developing anti-tumor drugs targeting gp130. Additionally, in tuberculosis (TB) patients, increased numbers of IL-12Rβ2^+^, WSX-1^+^, and gp130^+^ cells have been observed (98), suggesting that the IL-12Rβ2 and WSX-1/gp130 signaling pathways may play important roles in the immune response to TB, offering potential targets for novel therapeutic strategies.

This study not only establishes a comprehensive theoretical framework for elucidating the fundamental functions and mechanisms of these molecules across biological systems, but also provides critical scientific foundations for identifying more promising IL-12-related drug targets, developing precision immunotherapies, and advancing cross-species drug design. Furthermore, our findings highlight the importance of validating these targets in diverse species—a crucial step to ensure drug efficacy and safety across different biological systems. These insights may lead to breakthroughs in treating the aforementioned diseases while simultaneously offering novel strategies and directions for agriculture, aquaculture, and wildlife conservation.

### 4.3 Conservation and functional divergence

IL-12s and IL-12Rs exhibit remarkable evolutionary conservation across species, particularly in their key features including fn3 domains, Ig-like domains, and the three signature motifs. These conserved structural elements are essential for maintaining functional integrity and biological activity, underscoring their central role in immune regulation. Notably, despite these conserved structural characteristics, significant functional divergence has been observed, most prominently in the distribution and sequence conservation of IL-27p28 homologs. As a crucial component of the IL-27 cytokine, IL-27p28 is predominantly found in mammals where it displays exceptionally high sequence conservation. This restricted phylogenetic distribution suggests that IL-27p28 likely emerged relatively late in evolution compared to other IL-12 family members, potentially as an adaptive response to mammalian-specific immunological challenges. The evolutionary timing of IL-27p28’s emergence coincides with the development of more sophisticated adaptive immune systems in higher vertebrates. In this context, IL-27p28 may have evolved to fine-tune immune responses in these organisms.

### 4.4 Potential therapeutic targets and drug development

IL-12Rβ1 binds to the IL-12p40 through its N-terminal fn3 domain (1). A study identified D36, W37, and T38 as key amino acids for the interaction between p40 and IL-12Rβ1, particularly the W37K mutation, which significantly compromised IL-23-induced signaling and binding to IL-12Rβ1 (65) (**Figure 4**). Another study used bioinformatics analysis and site-directed mutagenesis to replace the basic amino acids arginine (R) and lysine (K) in the mouse IL-12p40 subunit cluster of amino acids 254-260 (RKKEKMK) with a neutral nonpolar alanine (A) (**Figure 4**), generating a mutant fusion protein named AAAEAMA. This mutant lacked heparin-binding activity but retained antigen-binding capacity and IL-12 biological activity (64). IL-12Rβ2 primarily interacts with the IL-12p35 subunit through its N-terminal Ig-like domain (1). The Y185R in mouse IL-12p35 and the Y189R in human IL-12p35 (**Figure 4**) can block binding to IL-12Rβ2 across species (63). These findings indicate that Y185 (mice) and Y189 (humans) are critical amino acids for the binding of IL-12p35 to IL-12Rβ2. In summary, these evolutionarily conserved residues and motifs across species have been demonstrated to critically influence receptor-ligand binding and complex activity, making them effective therapeutic targets for various immune disorders. The underlying mechanism lies in their essential role in modulating the structure and function of IL-12 family receptors, ligands, and receptor-ligand complexes, with particular emphasis on the pivotal contributions of these key amino acid residues and structural motifs.

Structural variations in IL-12 family members and their receptors significantly influence immune function and disease susceptibility. Certain single-nucleotide polymorphisms (SNPs) in IL12RB1, such as the R214-T365-R378 allele, are associated with increased tuberculosis risk (99), highlighting how receptor variants alter IL-12 signaling. Similarly, engineered IL-12 partial agonists retain the ability to induce IFNγ in CD8^+^ T cells while suppressing NK cell cytokine production (12). This cell-biased activity was confirmed *in vivo*, where such agonists triggered antitumor immunity against MC-38 adenocarcinoma without NK-mediated toxicity (12), showcasing the potential for structure-guided immune modulation. In autoimmune diseases like rheumatoid arthritis, structural variations in IL-12 family cytokines (e.g., IL-23-driven IL-17 cascade) disrupt the immune balance, affecting leukocyte migration, bone erosion, and angiogenesis, while IL-12, IL-27, and IL-35 exert counter-regulatory effects (100). In cancer, SNPs in IL12A and IL12B may alter protein expression or function, contributing to immune dysregulation and increased oncogenic risk (101). Combining these findings with our SeqLogo results (**Figure 4**), it is evident that, with their conserved cross-species sequence and spatial structural properties, these key amino acids are primarily found in the conserved structures that we have identified. The evidence above suggests that the key amino acid sites or structural features of IL-12s and IL-12Rs are widely conserved across animals, while some species-specific variations also exist. This implies that designing targeted drugs tailored to specific animal groups—primarily distinguished by Class—is feasible. Additionally, studying specific clusters of IL-12s and IL-12Rs proteins represents a promising new direction, as each cluster exhibits distinct characteristics from groups based on protein sequence features (**Figures 3C, F**).

### 4.5 Challenges and future directions

While this study provides valuable insights, several key challenges remain: (i) Cross-species analyses present substantial difficulties due to high genomic diversity (particularly in avian and piscine species) and limited data availability, complicating both ortholog identification and synteny analysis. (ii) Functional validation of conserved structural features requires genetic manipulation (e.g., knockout/overexpression) in appropriate animal models, yet the complexity of immune systems poses significant barriers to translating computational predictions into *in vivo* verification. (iii) Clinical translation faces bottlenecks as potential therapeutic strategies demand rigorous clinical trials—a time- and resource-intensive process requiring multidisciplinary collaboration. There is an urgent need to develop evolutionarily ancient model organisms (**Table 2**) that trace IL-12s and IL-12Rs origins, offering both cost-effective and ethically unconstrained research platforms. (iv) Structural prediction limitations persist; despite advances like ESMfold (58), the absence of crystallographic data for IL-12 family proteins (notably their four-helix bundles with fn3/Ig-like domains) in basal species introduces prediction artifacts.

Future priorities should focus on: (i) developing advanced bioinformatic tools for cross-species ortholog analysis, (ii) establishing functional validation models using CRISPR/Cas9-based genome engineering, (iii) prioritizing preclinical studies to bridge basic and clinical research, and (iv) exploiting ancient model organisms to circumvent ethical constraints while reducing costs. In summary, transitioning from evolutionary insights to clinical applications requires overcoming technical, validation, and translational hurdles, with the ultimate goal of developing novel immunotherapies.

## Data Availability

The original contributions presented in the study are included in the article/**Supplementary Material**. Further inquiries can be directed to the corresponding author.
