# Supplementary figures and images for: Evolutionary insights into Interleukin-12 family targets across 405 species
Source: Front Immunol. 2025 May 30;16:1584460. doi: 10.3389/fimmu.2025.1584460 (PMC12162339; doi:10.3389/fimmu.2025.1584460)

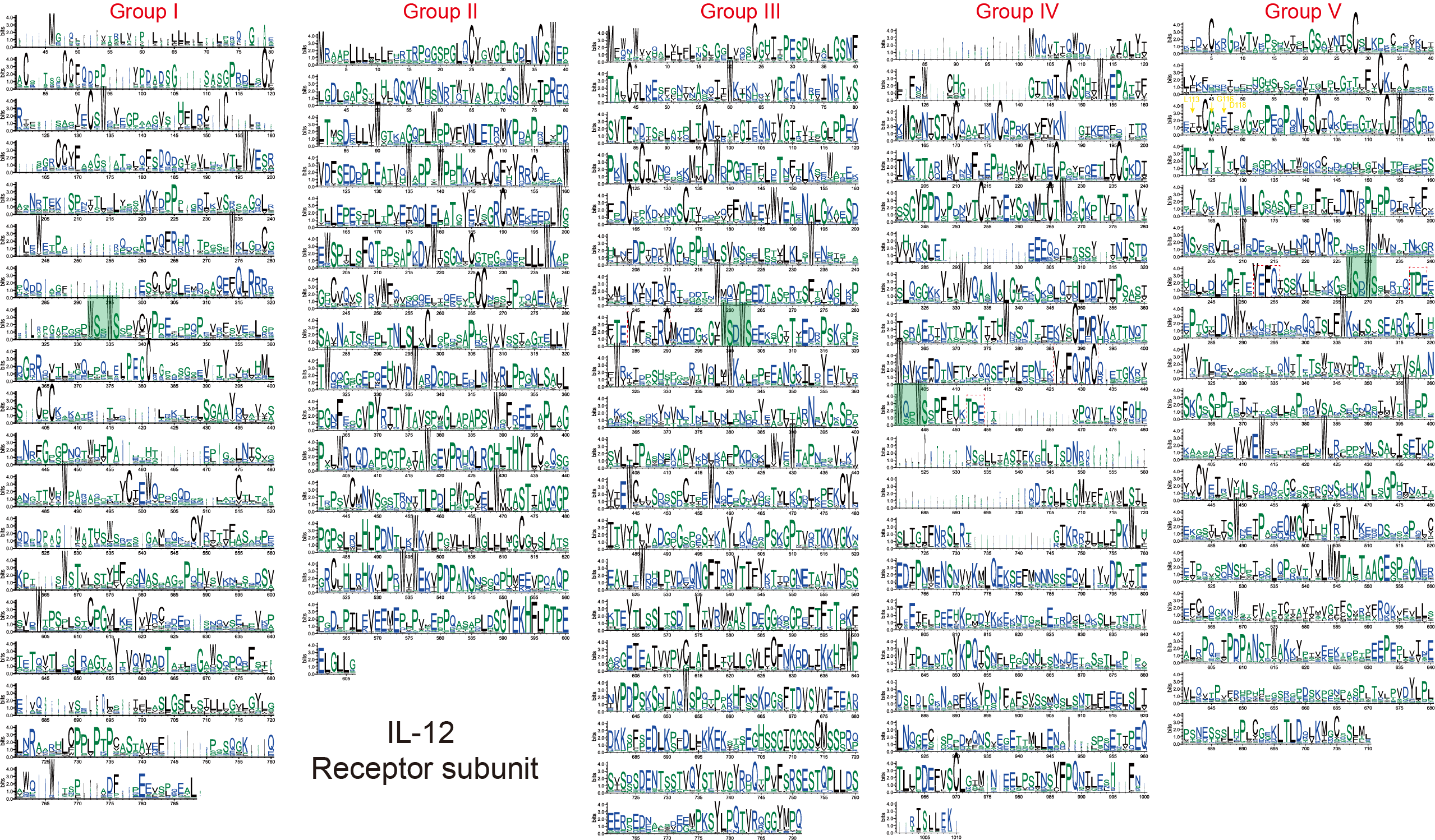

Supplement: Supplementary file 1 [file Image1.tif]

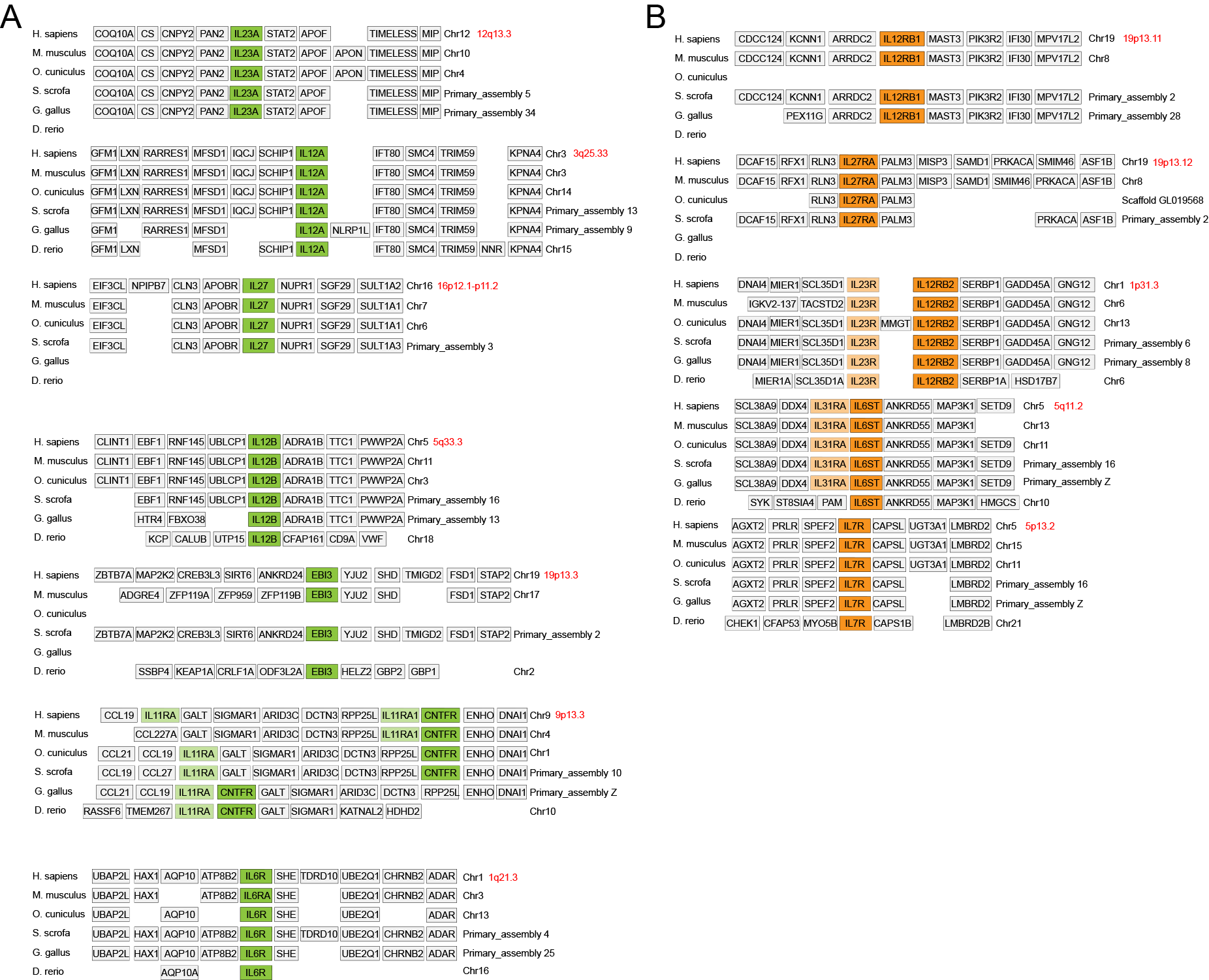

Supplement: Supplementary file 2 [file Image2.tif]
