# Supplementary material for: Evolutionary insights into Interleukin-12 family targets across 405 species
Source: Front Immunol. 2025 May 30;16:1584460. doi: 10.3389/fimmu.2025.1584460 (PMC12162339; doi:10.3389/fimmu.2025.1584460)
Supplement: Supplementary file 5 [file SupplementaryFile1.docx]

# Supplementary Figures and Tables

## Supplementary Figures

**Supplementary Figure 1.** Conserved features of IL-12Rs during animal evolution. SeqLogo plots of the five subgroups of IL-12Rs (trimmed), explaining the conserved sequence features of the five groups of IL-12Rs in more than 400 species of animals that have evolved to date.

**Supplementary Figure 2.** The genesis of IL-12s and IL-12Rs. **(A)**, **(B)** The distribution of IL-12s, IL-12Rs, and the genes that surround them in five model animals.

## Supplementary tables

**Supplementary Table 1.** Original data source and groups of IL-12s and IL-12Rs

**Supplementary Table 2.** Domains of IL-12s and IL-12Rs
